# The Uptake Mechanism of Cd(II), Cr(VI), Cu(II), Pb(II), and Zn(II) by Mycelia and Fruiting Bodies of *Galerina vittiformis*

**DOI:** 10.1155/2013/149120

**Published:** 2013-12-22

**Authors:** Dilna Damodaran, Raj Mohan Balakrishnan, Vidya K. Shetty

**Affiliations:** Department of Chemical Engineering, National Institute of Technology Karnataka, Surathkal, Srinivasnagar 575 025, India

## Abstract

Optimum concentrations of heavy metals like copper, cadmium, lead, chromium, and zinc in soil are essential in carrying out various cellular activities in minimum concentrations and hence help in sustaining all life forms, although higher concentration of these metals is lethal to most of the life forms. *Galerina vittiformis*, a macrofungus, was found to accumulate these heavy metals into its fleshy fruiting body in the order Pb(II) > Cd(II) > Cu(II) > Zn(II) > Cr(VI) from 50 mg/kg soil. It possesses various ranges of potential cellular mechanisms that may be involved in detoxification of heavy metals and thus increases its tolerance to heavy metal stress, mainly by producing organic acids and phytochelatins (PCs). These components help in repairing stress damaged proteins and compartmentalisation of metals to vacuoles. The stress tolerance mechanism can be deduced by various analytical tools like SEM-EDX, FTIR, and LC-MS. Production of two kinds of phytochelatins was observed in the organism in response to metal stress.

## 1. Introduction

Metal pollutants are released into the environment in many ways at potentially harmful levels [1]. In some areas, origin of heavy metals and metalloids in food chain is geological rather than anthropogenic; hence microorganisms in such areas develop effective strategy to cope with the harmful consequence of metal and metalloid exposures. Generally the heavy metal ions like Cd(II), Cr(VI), Cu(II), Pb(II), and Zn(II) are very reactive. Many of them possess high affinity especially for sulfhydryl groups in proteins and small biological molecules. As a consequence, the metal can critically affect function in many biological systems, as enzyme inhibitors or in other ways disturbing the pathways causing various lethal disorders.

Over the last decades, biosorption has emerged as a promising low cost methodology for the removal of metals from the environment, where biological components are employed to remove and recover heavy metals from aqueous solutions [2–4]. The metal removal mechanism is a complex process that depends on the chemistry of metal ions, cell wall compositions of microorganisms, physiology of the organism, and physicochemical factors like pH, temperature, time, ionic strength, and metal concentration [5]. The biosorption of heavy metals from soil process through phytoremediation is widely discussed. Researchers have studied phytoremediation thoroughly within last few decades and have understood that phytoremediation solely cannot solve all issues regarding heavy metal pollution because of its limitations like selectivity of plant, climatic inhibitions, tolerance to heavy metals, and back contamination by depuration or from ashes of fire woods; hence there is a need of a robust methodology which can go hand in hand with other techniques to remediate heavy metal contaminated areas more quickly, effectively, and economically.

Mushrooms or macrofungi can act as effective biosorbent alternative to plants in removing toxic metals from soil and the process is referred to as mycoremediation. Mushroom mycelia can serve as biological filters since their aerial structures consist of large biomass and have a tough texture which makes them potential sorbents [6]. Mushrooms are known to have high metal/metalloid tolerance which helps them to thrive and accumulate metals from the contaminated environment. They also have shorter life cycle (30 days) and better adaptability compared to plants; hence mycoremediation can be regarded as an evolved remediation technique. To understand and to engineer the bioaccumulation efficiency a thorough study on the mechanism of metal removal is essential.

Mushrooms response to metal stress in the environment by producing stress compounds of proteinous and nonproteinous origin. The cap of the mushrooms has been found to produce stress related factors which govern in metal ion uptake, that is, metallothionein glutothionine, and plastocyanin. Fungi have evolved metal tolerance and accumulation mechanism. Compared to microfungi, the mushrooms play a major role in accumulating heavy metals. Fruiting bodies of the mushrooms are considered to be advantageous to plants as they have shorter life cycle (30 days) and better adaptability compared to plants; hence mycoremediation can be regarded as an evolved remediation technique. The major factors governing the metal uptake are bioavailability and nature of soil. The bioavailability of metals is affected by numerous soil factors, such as cation exchange capacity [7], pH [8–10], and organic matter content [11, 12]. Understanding the mechanism of metal uptake from the soil to the fruiting bodies helps us to improvise the process by biotechnological tools; hence the study on the uptake mechanism plays a significant role in developing better remediation technique.

## 2. Materials and Methods

### 2.1. Bioaccumulation Studies

*Galerina vittiformis *spawns were used as the inoculums for *in vitro* fruiting body production. The spawns were mixed with soil mixture and were incubated at 23 ± 3°C in dark conditions. Casing of spawns was carried out in trays of 25 × 20 × 5 cm dimensions, sterilized with 70% alcohol. Soils used for the study were mixed with heavy metal (as PbNO_3_, CdSO_4_, CuSO_4_, K_2_Cr_2_O_7_, and ZnNO_3_) solutions to attain 50 mg kg^−1^ concentration and were dried in an oven. The dried soil was mixed with sawdust in the ratio of 3 : 1 (w/w). Sawdust increases the porosity and helps in better mushroom production. These soil mixtures were used for all bioaccumulation studies. Spawns were cased with the soil mixture and soil layer of about 3 to 4 cm thick was prepared. Cased trays were incubated in the dark conditions at 22 ± 2°C and 85 ± 5% relative humidity for a period of 25 days with periodical monitoring. At the end of the 25th day, the fruiting bodies formed were harvested using sterile forceps and allowed to dry at room temperature. 1 g of the dried biomass samples was mixed with 2 mL of 65% HNO_3_ and 6 mL of HCl and then digested in a microwave digester (CEM-MARS, USA) at 600 W for 20 min. The digested mixtures were cooled and were made up to 50 mL using deionized water. The cooled mixture is then filtered using Whatman No. 1 filter paper. These samples were analyzed for metal contents using atomic absorbtion spectrometer (AAS) [13, 14].

### 2.2. Mechanism of Bioaccumulation

To determine the tolerance and accumulation mechanism employed by mushrooms the fruiting bodies were analysed for primary and secondary stress components produced by them. The dried fruiting bodies and their extracts were analysed by various modern techniques, namely, scanning electron microscopy with energy dispersive X-ray analysis, fourier transforms infrared spectroscopy analysis, and liquid chromatography coupled with mass spectrometry to understand their metal uptake mechanisms.

#### 2.2.1. Scanning Electron Microscopy (SEM) with Energy Dispersive X-Ray Analysis (EDX)

In order to understand the role of surface activity on metal accumulation the toughs of fungal mycelia were subjected to SEM and EDX. The fungal mat obtained was harvested and dried in oven at 60°C. Theses dried biomasses were treated with 10% glutaraldehyde and then incubated for about 10–12 hours at 4°C. Further the biomass was treated with alcohol gradations (10%, 30%, 50%, 80%, and 100%) for 2 min to remove the water content [15, 28–30]. The pretreated specimens were then sputtered with gold particles using a sputter coater under vacuum and then observed under a scanning electron microscope (JSM-6380; JEOL, Tokyo) at an accelerating voltage of 12 or 15 kV to capture the images. EDX of these images was performed at 20 kV.

#### 2.2.2. Fourier Transforms Infrared Spectroscopy Analysis (FTIR)

The fruiting bodies and mycelia of *G. vittiformis* after the bioaccumulation studies were isolated and washed with distilled water and oven-dried at 60°C (Rotek, India). The dried biomass was then powdered and analyzed by Thermo Nicolet 6700, FTIR spectrometer to identify the functional groups and bonds present in them in response to heavy metal uptake which were responsible for the metal accumulation in cytosol.

To characterise the stress components produced in these biomass, FTIR was performed on fruiting body extracts. The stress components were extracted using Tris buffer system; 3 g of dried fruiting body was grounded using liquid nitrogen in a mortor and pestle; the homogenised extract was mixed with 3X Tris buffer (30 mM Tris, 250 mM NaCl, pH 7.6) in ice bath; centrifuged at 12,000 g for 15 min at 4°C; the supernatant was collected and stored at −20°C. The extract was then subjected to both FTIR and liquid chromatography coupled with mass spectra (LC-MS).

#### 2.2.3. Analysis of Stress Factors Using LC-MS

Fruiting body extracts were characterised using a liquid chromatographic column equipped with Accela pump and an Accela autosampler (Thermo Fisher Scientific, San Jose, CA, USA). Separation of analytes was conducted on a Luna PFP (2) analytical column (100 mm × 2.0 mm, 3 *μ*m). The LC mobile phases were (a) ammonium formate 0.75 mM adjusted to pH 3.5 with formic acid and (b) methanol. Separation was performed under isocratic conditions with 99% mobile phase A at flow rate of 200 *μ*L/min and a column temperature of 35°C. Total run time per sample was 10 min and all injection volumes were 10 *μ*L. Mass spectrometric analysis was performed using a TSQ Quantum Access (Thermo Fisher Scientific, San Jose, CA, USA) triple quadrupole mass spectrometer coupled with electrospray ionization (ESI) operated in multiple reactions monitoring (MRM) in positive mode. The MRM for GSH (*m/z* 308.1 → *m/z* 76.2 + 84.2 + 161.9) and GSSG (*m/z* 613.2 → *m/z* 230.5 + 234.6 + 354.8) were performed with collision energy optimized for each transition. The operating conditions for MS analysis were as follows: spray voltage, 2500 V; capillary temperature and voltage, 280°C and 35 V, respectively; Sheath gas and auxiliary gas flow, 30 and 5 arbitrary units, respectively; tube lens offset, 84 V for GSH and 115 V for GSSG. The mass spectrometer was employed in MS/MS mode using argon as collision gas. Data acquisition and analysis were performed with Xcalibur software, version 2.0 (Thermo Fisher Scientific, San Jose, CA, USA). 

## 3. Result and Discussions

### 3.1. Bioaccumulatation in Fruiting Bodies of Mushrooms

**Figure 1:**
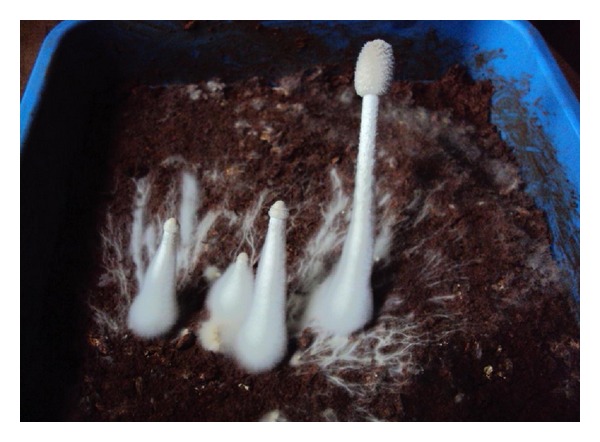
Fruiting body initials of organism *Galerina vittiformis* after 25 days of incubation in tray systems.

**Figure 2:**
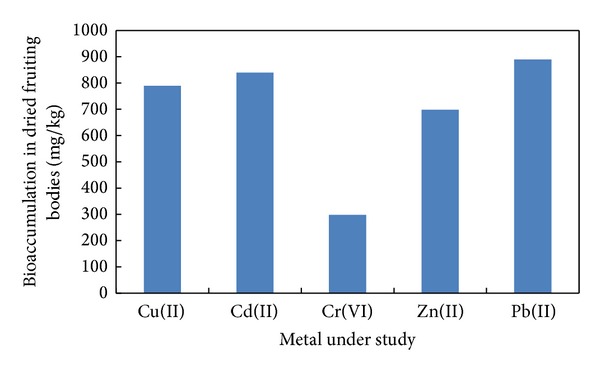
Bioaccumulation of metals by fruiting bodies of *Galerina vittiformis.*

Fruiting bodies of *Galerina vittiformis *produced after 25 days of incubation are shown in Figure 1. The results of bioaccumulation study are presented in Figure 2. The fruiting bodies act as the site of heavy metal accumulation and they can be easily separated from soil. This is considered to be advantageous over phytoremediation. In phytoremediation heavy metals are accumulated in plant parts like roots, twigs, and leaves and improper disposal methods increase the risk of back contamination. The levels of heavy metals accumulated in the fruiting bodies are shown in Figure 2 and are as follows: Cu(II) (800 mg kg^−1^), Cd(II) (852 mg kg^−1^), Cr(VI) (30 mg kg^−1^), Pb(II) (900 mg kg^−1^), and Zn(II) (700 mg kg^−1^). Thus the bioaccumulation potential of fruiting bodies of *G. vittiformis* was found to be in the following order: Pb(II) > Cd(II) > Cu(II) > Zn(II) > Cr(VI). Various researchers have found that organism's ability to accumulate heavy metals varies from species to species, at different stages of life cycle, amount of metal ion concentrations in the soil, nature of soil, and so forth [16, 21, 31–34].

The bioaccumulation potential of *G. vittiformis *was found to be higher than that of those mushroom species reported in the literature (summarized in Table 1). From Table 1 it is observed that nonedible mushroom species accumulate higher amounts of metal ions than the edible species. However, the bioaccumulation profile indicates that metal accumulation capability is species specific and mainly depends on its accumulation mechanism [20, 35–39]. 

### 3.2. Mechanism of Bioaccumulation

Heavy metals are known to act as a general protoplasmic poison, inducing the denaturation of proteins and nucleic acids [22, 40]. They can also break apart biological molecules into even more reactive species (such as reactive oxygen species) which will also disrupt biological processes. Hence only those species which can successfully tolerate these physiological stresses can successfully survive in heavy metal laden environment. Mushrooms respond to metal stress in the environment by producing stress compounds of proteinous and nonproteinous origin. The pileus (cap) of the mushrooms has been found to produce stress factors which help in sequestering the accumulated metal ions into their vacuoles. The most common stress components produced by plants and fungi are metallothionein (MT), glutothionine (GSH), phytochelatins (PCs), and plastocyanin [41]. From the studies of Inouhe et al. [42], Mehra et al. [43], Münger and Lerch [44], and Lerch [45], it is observed that macrofungi have evolved metal tolerance and accumulation mechanism compared to microfungi. Understanding the mechanism of the metal uptake from the soil to the fruiting bodies helps us to improvise the process by advanced molecular biology tools. Hence study on the uptake mechanism plays a significant role in developing better remediation technique. 

#### 3.2.1. Morphological Studies by Scanning Electron Microscopy (SEM) with Energy Dispersive X-Ray Analysis (EDX)

**Figure 3:**
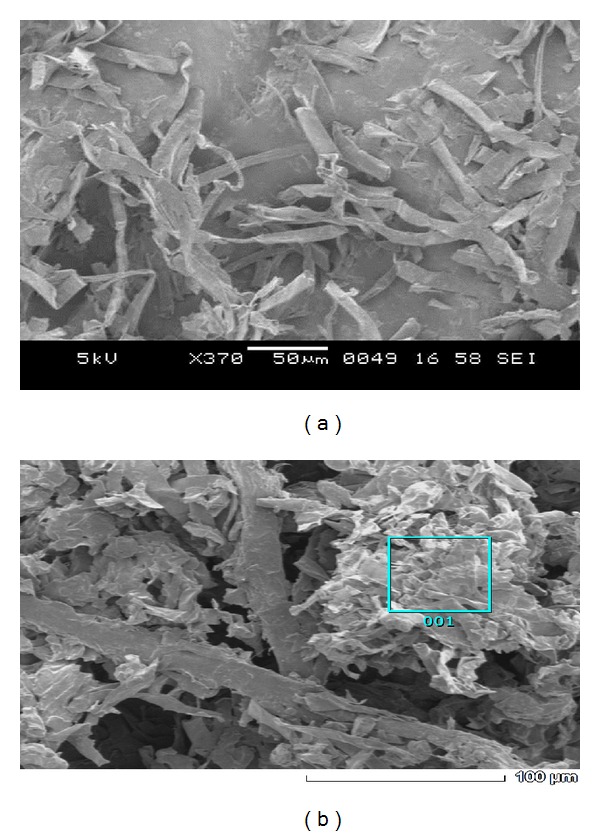
SEM images of organism *G. vittiformis* (a) untreated (b) Cd(II) treated (500–700X magnification).

**Figure 4:**
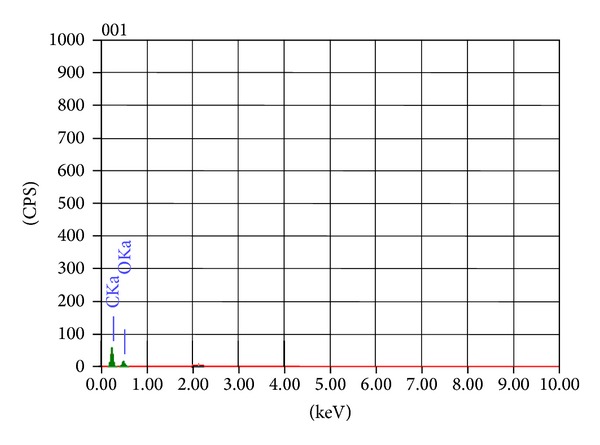
EDS analysis showing the metal content in *G. vittiformis* (control).

**Figure 5:**
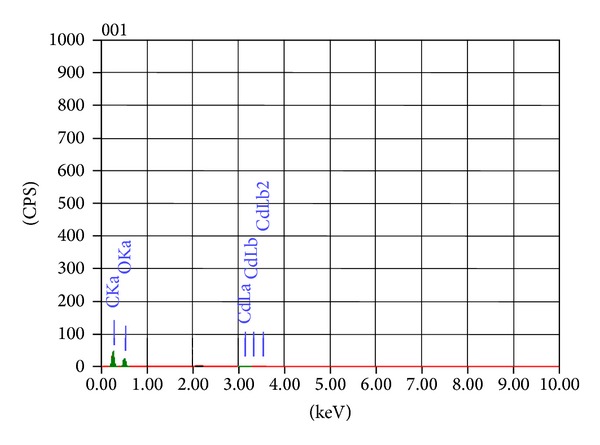
EDS analysis of organism *G. vittiformis* treated with Cd(II).

The effect of heavy metal on the morphology of *Galerina vittiformis* during the bioaccumulation process was studied through SEM image analysis.  Figure 3(a) revealed that the hyphae of *G. vittiformis* were cylindrical, septate, and branched before exposure to heavy metal. As shown in Figure 3(b) characteristic change in the morphology, curling, and formation of hyphal coils in response to Cd(II) stress is observed. Similar observations were made when exposed to other heavy metals. Cánovas et al. [46] reported that the surface of *Aspergillus *sp. also had rough texture due to protrusions on the hyphae on exposure to 50 mM of arsenate solution. Such modifications on the surface of fungi indicate the production of intracellular compounds due to heavy metals stress and result in increase in pressure within the mycelia leading to the outward growth of the cell wall structures [47]. Courbot et al. [48] have also observed that the impact of metal stresses had led to production of thiol compounds, especially GSH and MT due to intracellular detoxification of cadmium in the fungi, *Paxillus involutus. *According to them, the cell wall protrusions indicate increased formation of intracellular vacuoles that serve as storage compartments for thiol containing compounds. These compounds are responsible for the binding of metal ions into the intracellular regions and accumulate them in the vacuoles, thereby reducing their toxicity in the cytoplasm and improving tolerance levels. The energy dispersive spectroscopy (EDS) analysis was done to analyze the metal ionic concentrations in the mycelial surface indicating mycofilteration. The results of EDS analysis for the control mycelia and mycelium treated with Cd(II) are shown in Figure 4 and Table 2 and Figure 5 and Table 3, respectively. Only traces of Cd(II) were observed in EDS spectra whereas EDS of the mycelia exposed to other metals such as Cu(II), Pb(II), and Zn(II) showed no visible peaks for the metals (EDS spectra not shown), indicating the nondetectable levels of metals on the surface of the mycelia by SEM-EDS. Presence of traces or absence of metal peaks in the EDS spectra indicates that the metal removal by the mycelia of *Galerina vittiformis* may be attributed to vigorous intracellular bioaccumulation mechanism, rather than adsorption on the surface.

#### 3.2.2. Fourier Transform Infrared Spectroscopy (FTIR)

**Figure 6:**
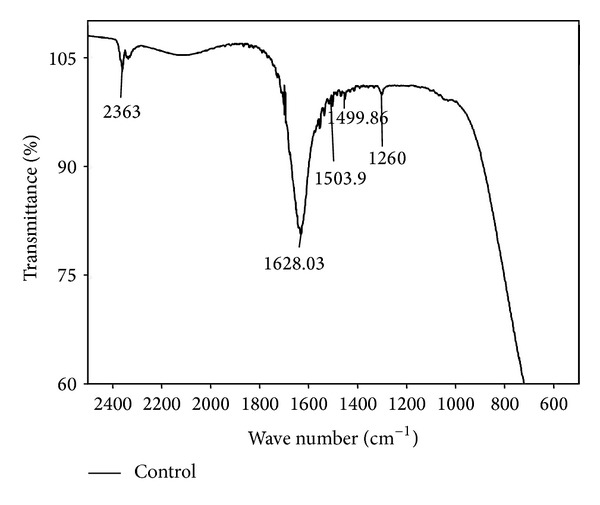
2D-FTIR results of *G. vittiformis* at metal free environment (control).

**Figure 7:**
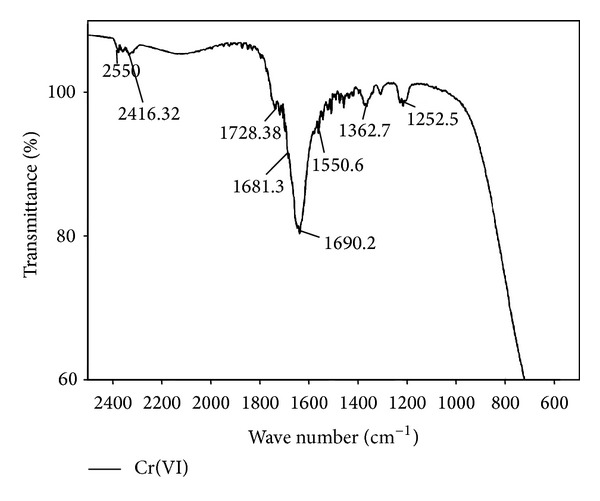
2D-FTIR results of *G. vittiformis* at Cr(VI) laden soil system.

**Figure 8:**
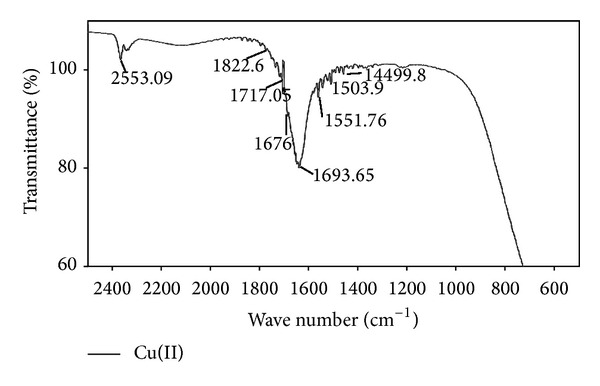
2D-FTIR results of *G. vittiformis* at Cu(II) laden soil system.

FTIR spectra of fruiting bodies extracts of *Galerina vittiformis* after bioaccumulation studies were analyzed to determine the presence and disappearance of any functional groups involved in metal accumulation mechanism. The FTIR spectrum was assessed by comparing the absorption bands of *G. vittiformis* grown on metal (Cu(II), Cd(II), Cr(VI), Pb(II), and Zn(II)) (50 mg/kg) contaminated soil to that of its spectrum obtained from control. Any changes in the finger print region, that is, 1000–1500 (amide-II and II regions) and 1500–1000 cm^−1^ (amide-III regions) which indicates the presence of higher amounts of acids, protenitous, and nonproteious compounds by *G. vittiformis* upon exposure to Cd(II), Cu(II), Pb(II), Cr(VI), and Zn(II). Each FTIR spectrum was studied thoroughly by comparing the peak values to their standard FTIR charts to determine the represented functional groups [32, 49–52]. The FTIR graphs obtained for all the studied metal ions are compared with control FTIR graphs to analyze the presence of stress factors produced during metal stress (Figure 6). The FTIR graphs of fruiting body obtained from Cr(VI) laden soil system showed the presence of stress related components like oxalic acid, that is, 1658 ± 5 and 1253 ± 5 and thiol group, that is, 2550 ± 5 (Figure 7) [53]. Similar peaks are also observed in the FTIR graphs of Pb(II) and Cd(II). Only thiol groups (2550 ± 5) are observed in Figure 8 for Cu(II) and Zn(II). Researchers like Qian and Krimm [54], Yang et al. [55], and Shi et al. [56] have used FTIR to detect the presence of both primary and secondary stress factors. FTIR gives only a preliminary identification of stress factors; hence the extracts are further subjected for LC-MS analysis to determine the components. Stress components present in the cellular extracts are further characterized by LC-MS analysis.

#### 3.2.3. Liquid Chromatography-Mass Spectrometry Analysis (LC-MS)

Metal homeostasis requires intracellular complexation of metals when there is a cellular surplus and later release of metals to metal requiring apoproteins. The excess metal ions are stored in the storage sites within the cell, for example, vacuoles [41]. LC-MS helps to identify those proteinous and nonproteinous metal ion trafficking components of *G. vittiformis *cells.

**Figure 9:**
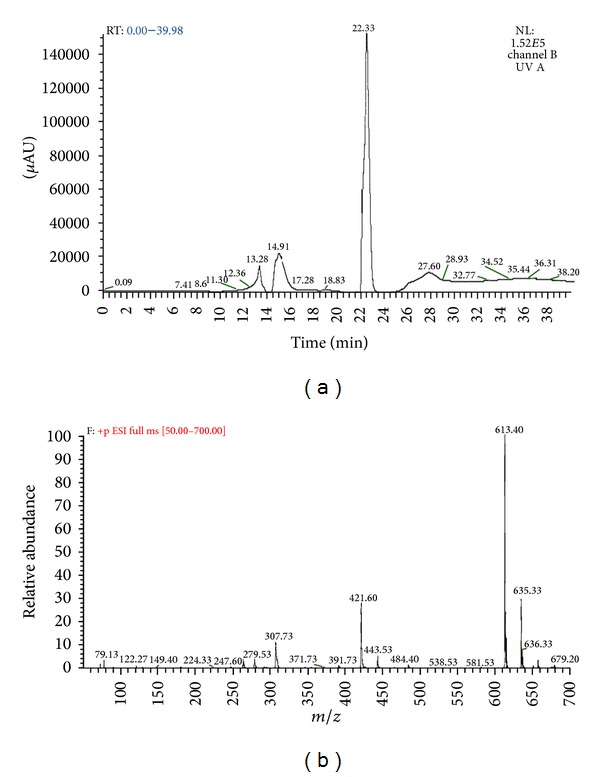
(a) Chromatogram produced by LC-MS analysis for Pb(II) at various retention times (min). (b) Chromatogram produced by LC-MS analysis for Pb(II) at various *m/z* ratios.

**Figure 10:**
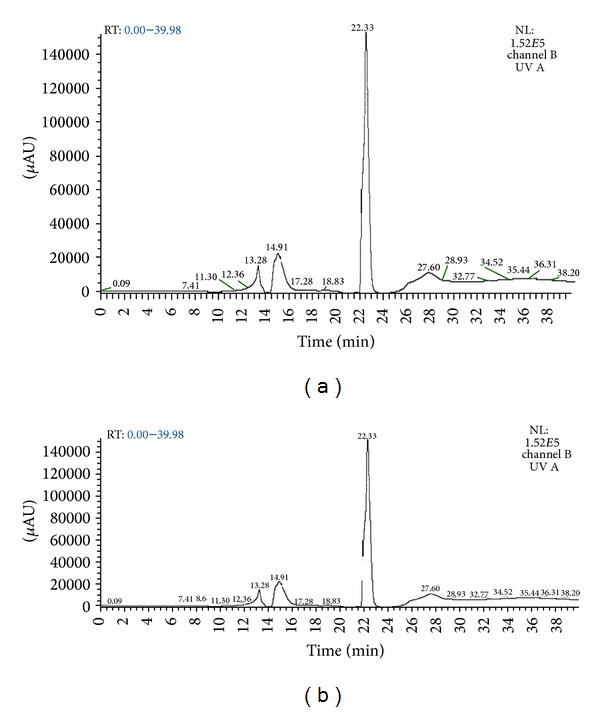
(a) Chromatogram produced by LC-MS analysis for Cu(II) at various retention times (min). (b) Chromatogram produced by LC-MS analysis for Cu(II) at various *m/z* ratios.

The LC-MS chromatograms for Pb(II) are shown in Figures 9(a) and 9(b). The data are obtained from Luna PFP (2) analytical column using ammonium formate and methanol as eluting buffers.  Figure 9(a) shows the retention time in minutes and Figure 9(b) shows the *m/z* ratio of each components present in fruiting body extracts. The peaks obtained in chromatograms were analyzed with the database to determine the components. On comparison with the literature it is observed that Figure 9(a) showed 2 peaks at 6–10 min retention, that is, 7.4 and 8.6 indicating the presence of cysteine (Cys) and glutamine (Glu) residues which are the subunits of phytochelatins (*γ*-glutamylcysteine).  Figure 9(a) also indicated 2 major peaks at 14–25 min retention time, that is, 14.9 and 22.3 indicated the presence of 2 types of phytochelatins (PC_2_ and PC_3_, resp.) while Figure 9(b) showed *m/z* peaks of glutathione (GSH), PC_2_, and PC_3_ at 307, 538 and 679, respectively. Similar kinds of chromatograms obtained for both Cd(II) and Cr(II) indicate the presence of phytochelatins (PCs). Figures 10(a) and 10(b) show the chromatogram of Cu(II) indicating the presence of both PC_2_ and PC_3_ (539 and 679 *m/z* peaks) [56–59].

**Figure 11:**
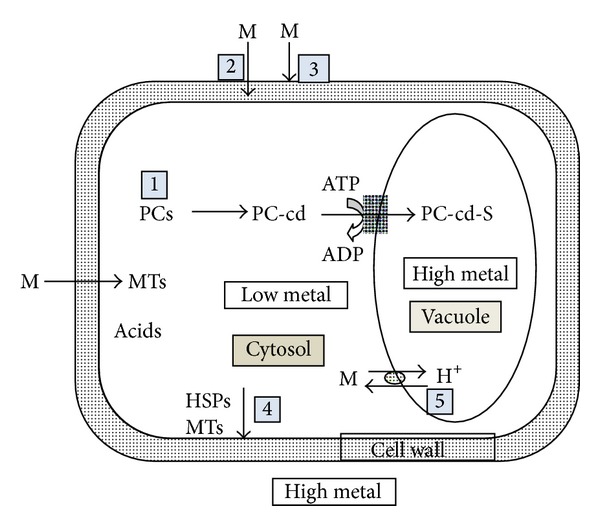
Schematic representation of the proposed mechanism of metal uptake by *Galerina vittiformis.* (1) Metal adsorption on fungal mycelial surface which acts as roots of fruiting bodies. (2) Uptake and storing in periplasmic space passive absorption. (3) PC and acid production in response to metal stress. (4) Acids act as HSPs (heat shock proteins) that bind to metal and store them to periplasmic space. (5) Transport and accumulation of metals in vacuole.

From the studies of Grill et al. [60], Gekeler et al. [61], Liedschulte et al. [62], and Gill and Tuteja [63], it was revealed that the Phytochelatin of the general formula (*γ*-Glu-Cys)_*n*_ is the principal heavy metal detoxifying component in both plant and fungal kingdom. The phytochelatins can be viewed as linear polymers of the *γ*-glutamylcysteine (*γ*-Glu-Cys) portion of glutathione. These peptides could be enzymatically produced by stepwise condensation of *γ*-Glu-Cys moieties to growing phytochelatin chain (PC). The PC plays a key role in maintaining cell homeostasis under heavy metal stress by binding to heavy metals like Cd, Zn, Cr, and so forth and trafficking them to vacuoles or periplasmic space for storage [63]. Hence from the result of the present study, the mechanism of metal accumulation can be summarised in Figure 11. Similar heavy metal accumulation mechanism of PC was reported in various metal resistant plants and algal species [41, 57, 60, 64–70].

## 4. Conclusion

Removal mechanism of Cu(II), Cd(II), Cr(VI), Pb(II), and Zn(II) metals from contaminated soil using a macrofungus, *Galerina vittiformis, *was investigated in *in vitro* system.

- *G. vittiformis* shows efficient bioaccumulation potential in removing most potent heavy metals unlike other known organisms.
- Trace amounts of metal ions are observed in SEM-EDX in the surface indicating primary adsorption step in accumulation.
- FTIR spectral analysis indicates the presence of secondary stress components like organic acids, phytochelatins, and so forth.
- The subunits of phytochelatin chain (PC); that is, Glu-Cys moieties are observed in LC-MS analysis.
- *G.vittiformis* produced two types of Phytochelatins, namely, PC_2_ and PC_3_ in response to Cu(II), Cd(II), Cr(VI), Pb(II), and Zn(II) metal stress.
- Phytochelatins are known to transfer the excess metal ions to the vacuole of the cell, thereby reducing the cellular toxicity.

Hence from analysis reports of SEM-EDX, FTIR, and LC-MS, phytochelatin plays a major role in removing heavy metal from soil by *G. vittiformis*. The study of mechanism is significant as any modifications in the gene regulating phytochelatin production can be modified to increase the metal accumulation ability. Mycoremediation can be considered as an alternative to other known methods in heavy metal removal from the soil owing to its short action period and better accumulation efficiency. Integration of this technology with advanced agronomical and engineering skills can transform mycoremediation as a competitive remediation tool.

## Figures and Tables

**Table 1:** Heavy metal content in fruiting body (sporocarp) of various mushrooms.

Sl. number	Mushroom species	Metal content in sporocarp, mg kg^−1^ of dry wt.	References
1	*Agaricus bisporus* ^ 1^	Pb (4), Cd (3.48), Cu (5.)	[15]
	*Boletus edulis* ^ 1^	Cu (66.4), Cd (6.58), Pb (3.03)	
	*Lepiota rhacodes* ^ 2^	Pb (66), Cd (3.7)	
	*Paxillus rubicondulus* ^ 1^	Pb (0.69), Cd (0.78), Cu (51.0) Zn (16.8)	

2	*Agaricus bisporus* ^ 1^	Cu (107), Pb (1), Zn (57.)	[16]

3	*Helvella leucomelaena* ^ 2^	Pb (4.8), Cd (.0)	[17]
	*Pleurotus* sp. ^1^	Pb (3.4), Cd (1.18), Cu (13.6), Zn (9.8)	

4	*Tricholoma terreum* ^ 1^	Cu (5), Zn (179), Cd (0.56), Pb (4.4)	[18]
	*Helvella leucomelaena* ^ 2^	Pb (3.1), Cd (1.1)	

5	*Paxillus involutus* ^ 2^	Cu (57.0), Pb (1.6.0), Fe (991), Cd (0.84), Pb (3)	[19]
	Rhizopogonaceae *luteolus* ^1^	Cu (13), Zn (30), Mn (13), Fe (620), Cd (0.26), Pb (2.8).	
	*Omphalotus olearius* ^ 2^	Cu (21), Zn (27), Mn (36), Fe (95), Cd (1.3), Pb (5.2).	
	*Hygrophorus hedyricii* ^ 2^	Cu (37), Zn (97),Mn (11), Fe (395), Cd (1.2), Pb (2.7)	
	Ciocybe dealbata^2^	Cu (41), Zn (115), Mn (30), Fe (386), Cd (0.86), Pb (3.2)	
	*Lepiota alba* ^ 2^	Cu (29), Zn (86), Mn (22), Fe (779), Cd (0.8), Pb (5.8)	

6	*Tricholoma terreum* ^ 2^	Pb (4), Cd (1.6), Cu (35.8), Zn (48.0)	[20]
	*Agaricus bisporus* ^ 1^	Pb (0.8), Cd (0.78)	

7	*Pseudevernia furfuracea* ^ 2^	Al (12.51), As (0.23), Cd (0.19), Cu (2.5), Cr (0.11), Pb (5.1), Zn (17.9), Mn (12.9)	[21]
	Scorpiurus circinatum^2^	Al (17.51), As (0.32), Cd (0.35), Cu (3.2), Cr (1.1), Pb (6.3), Zn (46.1), Mn (46.7)	

8	*Aspergillus foetidus* ^ 2^	Al (32.5), Co (5.95), Cr (6.23), Mg (44.9), Zn (2.4), Ni (189.5)	[22]

9	*Poria* sp.^2^	Zn (90.3), Cu (30.8), Pb (1.0), Mn (31.3), Cd (0.1)	[23]
	*Nectria cinnabarina* ^ 1^	Zn (30.1), Cu (29.3), Pb (1.9), Cd (0.2), Mn (19.3)	
	*Ganoderma lucidum* ^ 1^	Zn (60.1), Cu (43.8), Pb (0.7), Mn (30.4), Cd (0.31)	
	Paragyrodous sphaerosporous^1^	Zn (115), Cu (34.4), Pb (0.4), Mn (37.3), Cd (0.2)	
	*Polyporus frondosus* ^ 1^	Zn (120.1), Cu (34.4), Pb (0.4), Mn (37.3), Cd (0.2)	

10	*Phellinus badius* ^ 2^	Cd (110), Cu (60), Hg (61), Ni (56)	[3]
	*Phellinus sanguineus* ^ 2^	Cd (80), Cu (42), Hg (35), Ni (66)	

11	*Tricholoma terreum* ^ 2^	Pb (3.64), Cu (34.86), Cd (0.67), Zn (54.13), Cr (2.54)	[24]
	*Boletus badius* ^ 1^	Cu (44.54), Pb (4.48), Cd (0.91), Zn (34.17), Fe (264.62), Cr (2.86)	
	*Russula delica* ^ 1^	Cu (19.55), Pb (2.02), Cd (1.22), Zn (38.5), Cr (6.95)	

13	Pleurotus platypus^1^	Cd (34.9), Pb (27.10)	[25]
	*Agaricus bisporus* ^ 1^	Cd (33.7), Pb (29.67)	

14	*Lactarius deliciosus* ^ 1^	Cd (0.26), Cr (0.12), Cu (6.15), Pb (0.73), Zn (76.7)	[26]
	*Rhizopogon roseolus* ^ 1^	Cd (0.18), Cr (0.10), Cu (21.2), Pb (2.03), Zn (36.7)	
	*Russula delica* ^ 1^	Cd (0.42), Cr (0.27), Cu (52.2), Pb (0.77), Zn (58.2)	

15	*Sarcosphaera crassa* ^ 1^	Ag (0.044), As (8.03), Cd (0.016), Cr (0.98), Pb (0.02)	[27]
	*Cantharellus cibarius* ^ 1^	Ag (0.022), As (0.03), Cd (0.036), Cr (0.69), Pb (0.04)	
	Suillus luteus^1^	Ag (0.015), As (0.15), Cd (0.034), Cr (0.15), Pb (0.06)	
	*Morchella rigida* ^ 1^	Ag (0.087), As (0.24), Cd (0.007), Cr (0.44), Pb (0.02)	
	*Agrocybe aegerita* ^ 1^	Ag (0.074), As (0.44), Cd (0.010), Cr (0.25), Pb (0.018)	

15	***Galerina* sp.** ^ 2^	**Cd** (**85**), **Pb** (**900**), **Cu** (**800**), **Zn** (**700**), **Cr** (**30**)	

^1^Edible, ^2^Nonedible.

**Table 2:** EDS quantitative analysis of organism *G. vittiformis* (control).

Element	KeV	Mass%	Error%	At%	K
C K	0.277	59.75	0.17	66.41	68.8722
O K	0.525	40.25	1.08	33.59	31.1278

Total		100		100	

**Table 3:** EDS quantitative analysis of organism *G. vittiformis* treated with Cd(II).

Element	keV	Mass%	Error%	At%	K
C K	0.277	50.25	0.15	57.62	53.19
O K	0.525	49.15	0.72	42.30	45.850
Cd L	3.132	0.60	0.72	0.07	0.9590

Total		100		100	
